# Total Internal Reflection Ellipsometry Approach for Bloch Surface Waves Biosensing Applications

**DOI:** 10.3390/bios12080584

**Published:** 2022-07-30

**Authors:** Ernesta Bužavaitė-Vertelienė, Vincentas Maciulis, Justina Anulytė, Tomas Tolenis, Algirdas Baskys, Ieva Plikusiene, Zigmas Balevičius

**Affiliations:** 1 State Research Institute Center for Physical Sciences and Technology, Saulėtekio Ave. 3, LT-10257 Vilnius, Lithuania; ernesta.verteliene@ftmc.lt (E.B.-V.); vincentas.maciulis@ftmc.lt (V.M.); justina.anulyte@ftmc.lt (J.A.); tomas.tolenis@ftmc.lt (T.T.); algirdas.baskys@ftmc.lt (A.B.); 2 Faculty of Electronics, Vilnius Gediminas Technical University, Naugarduko St. 41, LT-03227 Vilnius, Lithuania; 3 NanoTechnas—Centre of Nanotechnology and Materials Science, Faculty of Chemistry and Geosciences, Vilnius University, Naugarduko Str. 24, LT-03225 Vilnius, Lithuania

**Keywords:** Bloch surface waves, total internal reflection ellipsometry, bovine serum albumin, biosensing

## Abstract

A one-dimensional photonic crystal with an additional TiO_2_ layer, supporting Bloch surface waves (BSW), was used for enhanced signal sensitivity for the detection of protein interaction. To compare the optical response of BSW and photonic crystals (PC), bovine serum albumin and specific antibodies against bovine serum were used as a model system. The results obtained show the enhanced sensitivity of p- and s-BSW components for the 1D PC sample with an additional TiO_2_ layer. Furthermore, a higher sensitivity was obtained for the BSW component of p-polarization in the PC sample with an additional TiO_2_ layer, where the sensitivity of the ellipsometric parameter Ψ was five times higher and that of the Δ parameter was eight times higher than those of the PC sample. The capabilities of BSW excitations are discussed from the sensitivity point of view and from the design of advanced biosensing.

## 1. Introduction

Metal oxides are widely used in various sensor applications [1,2,3,4,5] due to their optical transparency and tolerance to mechanical stress. Optical and electrical properties of such materials can be altered by modifying their structure, size and composition [6,7]. Metal oxides are suitable and widely applied dielectric materials for the production of various porous nanostructures [8,9,10] and photonic crystals (PC), due to the possibility of fabricating periodic nanostructures with high and low refractive index values [11,12,13,14]. Moreover, such periodic dielectric materials on a wavelengths scale comparable with period can generate Bragg reflections for incident photons and also can be used for the excitation of Bloch surface waves (BSW). The BSWs are a type of surface electromagnetic waves (SEW) generated at the interface between periodic dielectric multilayered structure and surrounding media [15] in which the electric field decays exponentially at the PC/ambient interface. Compared to the widely used surface plasmon resonance (SPR) generated on thin metal films, which has a narrow range of noble metals to choose from, the BSW excitation can be tuned in a wide spectral range by changing the materials and thickness of the bilayers in the 1D PC [16,17]. In recent studies, Lereu [18], Sinibaldi [16] and Balevicius [19] reported that BSW excitation has a higher sensitivity to amplitude changes than SPR. Similar to SPR, BSWs have been used for various nanophotonic applications [20,21,22]; however, such electromagnetic surface waves distinguished different properties from the surface plasmon polaritons. The propagation losses of BSWs are lower than those of SPPs due to purely dielectric materials, leading to higher quality factors of such surface resonances.

BSW excitation can be generated in both p- and s-polarizations, which allows the monitoring of both polarization states as the traditional SPR can only be generated in p-polarization [10]. To generate the BSW, a total internal reflection (TIR) geometry has to be used, as the dispersion of BSW lies below the light line in vacuum; thus, in order to generate BSW, the PC is attached to the prism base. The BSW can only be excited in the PC photonic band gap at a specific range of angles of incidence (AOI) due to the matching of the BSW in-plane wave vector. Additionally, the utilization of TIR prevents light travelling directly through liquids, which would otherwise reduce the signal intensity [23].

For a detailed analysis of BSW polarization states, the spectroscopic ellipsometry (SE) method in total internal reflection configuration (TIRE) can be applied [24]. SE allows us to measure not only the light polarization of p- and s-amplitude changes (Ψ), but also the phase difference (Δ), increasing the sensitivity of the TIRE method [25] compared to SPR reflection intensity measurements. The SE method also allows us to perform real-time measurements, enabling the measurement of the interaction between biomolecules [26,27,28]. BSWs have been investigated as a potential application for optical biosensing [17,29]. Due to the low extinction coefficient in dielectrics, the electromagnetic field does not quench in dielectrics, compared to metals, and thus, BSWs produce narrower resonance than SPR [16,18,19]. Moreover, dielectrics are biocompatible with biomolecules, making BSWs an attractive tool for optical biosensor application [30].

The aim of this research was to compare the optical response and sensitivity properties of two 1D photonic crystals with and without an additional TiO_2_ layer. Employing the TIRE method, the total internal reflection conditions provide the possibility of generating Bloch surface waves at the interface of the 1D PC and the ambient environment. Dispersion relations in liquid ambient were compared for both p- and s-polarizations in order to evaluate the sensitivity of different structures supporting BSW. Protein immobilization was performed to compare these BSW-based samples for possible applications in biosensing.

## 2. Materials and Methods

### 2.1. Materials

(3-Aminopropyl)triethoxysilane (APTES, 99%), N-(3-dimethylaminopropyl)-N′-ethylcarbodiimide hydrochloride (≥98%, EDC), N-hydroxysuccinimide (98%, NHS) and sodium dodecyl sulphate (≥99%, SDS) were purchased from Sigma-Aldrich (Taufkirchen, Germany). Bovine serum albumin (BSA, fraction V), phosphate buffered saline (PBS) tablets (0.14 M NaCl, 2.7 mM KCl, 10 mM phosphate buffer, PH-value 7.4) and sodium hydroxide (99%, NaOH) were obtained from Carl Roth GmbH&Co (Karlsruhe, Germany). Affinity-purified anti-bovine serum albumin antibodies (anti-BSA) were purchased from Immunology Consultants Laboratory, Inc. (Portland, OR, USA). All aqueous solutions were prepared in deionized water.

### 2.2. Formation of PC and PC/TiO_2_

**Figure 1:**
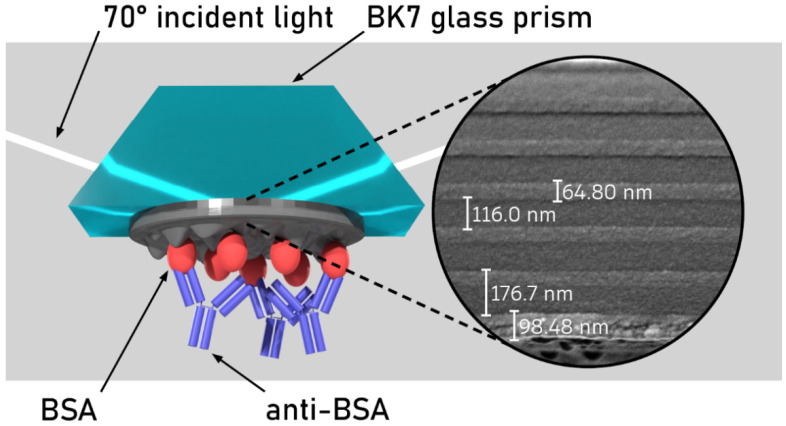
Experimental setup and SEM micrographs of the PC/TiO_2_ sample formed using six bilayers of ~116 nm SiO_2_ and ~65 nm TiO_2_ and a 98.48 nm nanoporous TiO_2_ layer on the top of the structure for the Bloch surface wave (BSW) excitation.

Two samples with a photonic crystal structure (PC) consisting of 6 TiO_2_/SiO_2_ bilayers and PC covered by a nanoporous TiO_2_ layer (PC/TiO_2_) were formed on a BK7 glass slide substrate using an ion beam sputtering (IBS) method. The porous TiO_2_ layer was formed by the glancing angle deposition (GLAD) method. Before the formation of PC and PC/TiO_2_ structures on glass, the vacuum chamber was held at 50 °C for 1 h. For the removal of the impurity layer, the ion source was used for the pre-sputtering of the target before deposition [31]. During the removal process, oxygen gas was supplied to the substrate for the complete oxidation of the formed layers. A radio frequency grid system-based ion source was used to bombard a flat metal target, consisting of high-refractive index (HI) and low-refractive index (LO) materials at a set angle of incidence of 57°. Typical deposition speeds for LO and HI refractive indices were 1 Å/s and 0.6 Å/s, respectively. The HI and LO refractive index targets were swapped with a linear translation stage. The substrates holding the circular rack were rotated around the axis at a speed of 20 rpm. The structures formed, PC and PC/TiO_2_, were characterized by using scattering electron microscopy (SEM), with a dual beam Helios Nanolab 650 (FEI) (Oxford Instruments, Abingdon, UK). The SEM micrographs presented in Figure 1 show that the formed PC consisted of six bilayers with TiO_2_ d = 64.8 nm and SiO_2_ d = 116 nm. The PC/TiO_2_ sample consisted of the same PC, and it was covered by a 98.48 nm porous TiO_2_ layer on top.

### 2.3. TIRE Measurements

The ellipsometric measurements were performed using a J.A. Woollam (J.A. Woollam Co., Inc., Lincoln, NE, USA) M2000X ellipsometer with a rotating compensator. The TIRE experiments were conducted at a 70° angle of incidence in the 200 nm–1000 nm wavelength range. The glass slide with the formed PC structure was attached to a 70° angle BK7 glass prism using a refractive index matching liquid and placed on a Teflon liquid handling chamber, where liquid intake was controlled by a connecting valve to which a syringe with solution is connected. The ellipsometric parameter spectra of Ψ(λ) and Δ(λ) were registered in situ at the rate of one spectrum in 4 s. Ellipsometry data were analyzed using the proprietary software Complete EASE (J.A. Woollam, Lincoln, NE, USA). The ellipsometric parameter Ψ corresponds to the reflected light wave p- and s-polarized amplitudes ratio and Δ is the p- and s-polarization difference of the light wave upon the light’s reflection from the sample surface.

### 2.4. Functionalization of PC/TiO_2_ Surface for Covalent BSA Immobilization

Both samples, (i) PC and (ii) PC/TiO_2_ with a top layer of TiO_2_, were modified by means of APTES. The functionalization of the surfaces with APTES was performed in the vapor phase. In this process, 0.5 mL of APTES was deposited in a small vial, then placed in a glass vessel along with glass slides with deposited PC nanostructures. To minimize contact with air, argon gas was pumped inside the glass vessel and closed. The glass vessel was placed in a 90 °C oven and kept overnight. Afterwards, the modified PCs were washed with ethanol and then dried in an oven at 110 °C for 15 min. During this step, the surfaces of PCs were functionalized with amino groups, which was necessary for the covalent immobilization of BSA.

### 2.5. Covalent BSA Immobilization and Formation of BSA/Anti-BSA Complex

For covalent immobilization of BSA on (i) PC and (ii) PC/TiO_2_, the PBS solution was injected into the chamber and was left for 45 min to establish a baseline. During that time, the activation of BSA was conducted. In a vial, 667 µL of 0.4 M EDC, 667 µL of 0.1 M NHS, 447 µL of PBS and 200 µL of 1 mg/mL BSA were mixed and left to activate BSA carboxyl functional groups for 30 min. Subsequently, the final solution containing 0.1 mg/mL BSA was injected into the chamber and then left for 60 min; after that, the PBS solution was injected to wash any non-covalently immobilized BSA proteins. For the formation of the BSA/anti-BSA immune complex, a solution of 0.05 mg/mL anti-BSA antibodies diluted in PBS was injected into the chamber for 80 min. Then, it was rinsed with PBS for 15 min to wash any non-specifically bounded antibodies.

## 3. Results and Discussion

**Figure 2:**
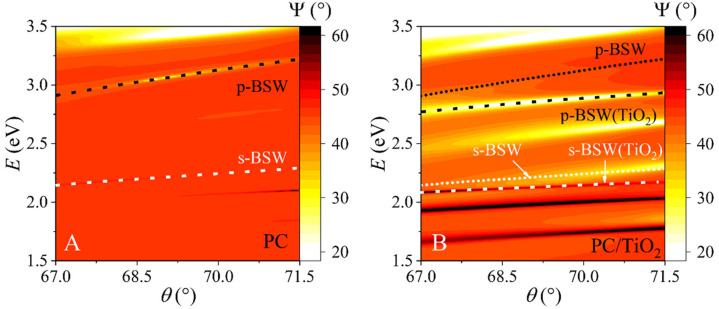
Dispersion relations of Bloch surface waves (BSW) for PC (**A**) and PC/TiO_2_ samples (**B**). The p-BSW and s-BSW mark the p- and s-polarization components of the BSW excitation. The dashed lines in (**B**) marked as p-BSW and s-BSW represent the BSW excitations of the p- and s-polarization components, respectively, obtained from the PC (**A**) sample.

The measured ellipsometric parameters Δ(λ) and Ψ(λ) were expressed as the p- and s-polarized intensity, using the data acquisition software Complete EASE (J.A. Woollam Co., Inc., Lincoln, NE, USA). The ellipsometric parameter Ψ maps depending on the energy (E) vs. angle of incidence (AOI) are presented in Figure 2A,B. The Ψ parameter represents the ratio between the p- and s-polarization components of the dispersion relations of investigated Bloch surface wave (BSW) excitations.

The BSW excitations are marked as black and white dotted lines in Figure 2A,B for the p- and s-polarization components, respectively. For a 1D PC sample, the BSW of p-BSW and s-BSW polarizations are excited at 3.12 eV and 2.24 eV, respectively, at 70° AOI. For the 1D PC/TiO_2_ sample, the components are at 2.88 eV and 2.14 eV for p-BSW and s-BSW, respectively. As can be seen from Figure 2B, the 1D PC/TiO_2_ structure has more vivid BSW excitation components than the PC (Figure 2A) structure, and the p-BSW and s-BSW components shift to longer wavelengths, showing that the BSW excitations can be easily tuned in the spectra.

### Application of PC/TiO_2_ Structure for Biosensing

**Figure 3:**
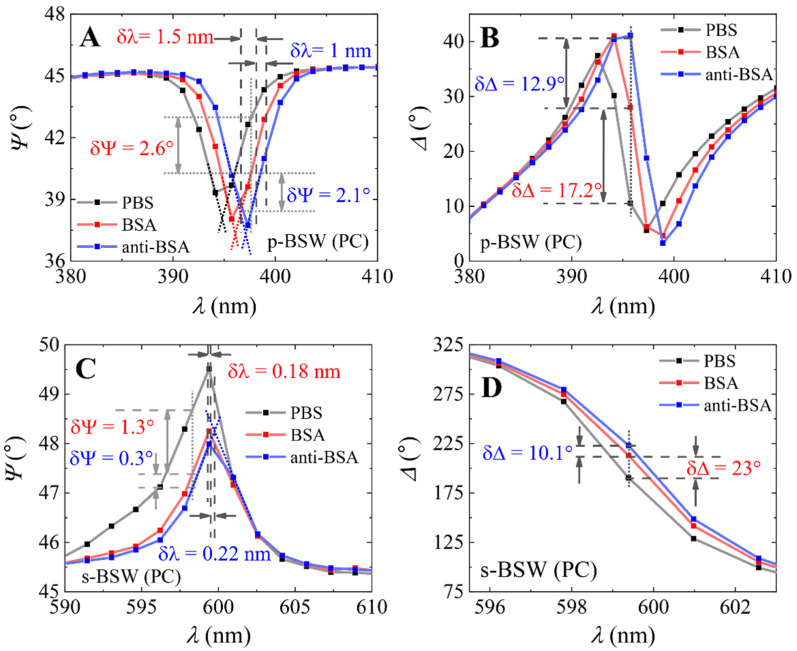
Spectra of ellipsometric parameters Ψ(λ) and Δ(λ) of BSW obtained on PC sample for PBS (black curve), BSA (red curve) and anti-BSA (blue curve); (**A**,**B**) for p-polarization and (**C**,**D**) for s-polarization.

**Figure 4:**
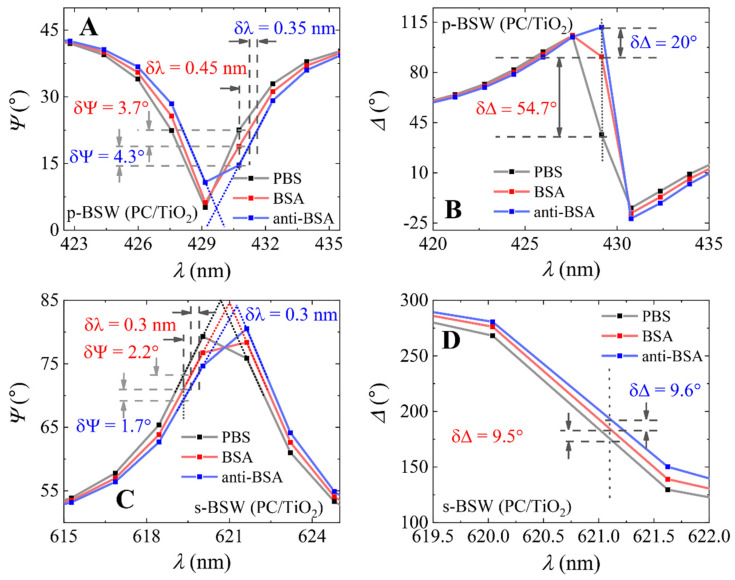
Spectra of ellipsometric parameters Ψ(λ) and Δ(λ) of BSW obtained on the PC/TiO_2_ sample for PBS (black curve), BSA (red curve) and anti-BSA (blue curve); (**A**,**B**)—for p-polarization and (**C**,**D**) for s-polarization.

The covalent immobilization of BSA and affinity interaction with anti-BSA (complex formation of BSA/anti-BSA) were investigated on both samples: (i) PC and (ii) PC/TiO_2_ samples. As can be seen from Figure 3 and Figure 4, both p- and s-polarized light can be used for the analysis of BSA immobilization and BSA/anti-BSA complex formation. Figure 3 shows the spectra of the ellipsometric parameters Ψ(λ) and Δ(λ) for PBS baseline (black curve), after covalent immobilization of BSA on the silanized PC surface (red curve), and after BSA/anti-BSA immune complex formation (blue curve). The spectral shift (δλ) and changes δΨ and δΔ to the ellipsometric parameters Ψ and Δ of the BSW (p- and s-polarization components) were evaluated for both PC and PC/TiO_2_ samples at 70° AOI (Figure 3 and Figure 4). For the PC sample, the change in parameter Ψ (Figure 3A,C) from PBS to anti-BSA was δΨ_p-BSW_ = 4.7° and δΨ_s-BSW_ = 1.6° for the p- and s-polarization components of BSW excitation, respectively. The spectral shift was equal to δλ_p-BSW_ = 2.5 nm and δλ_s-BSW_ = 0.4 nm. As can be seen from Figure 3B,D, the Δ parameter change from PBS to anti-BSA was δΔ_p-BSW_ = 30.1° and δΔ_s-BSW_ = 33.1° for the p- and s-polarization components, respectively.

The same curves for PBS, BSA covalent immobilization and BSA/anti-BSA complex formation were obtained on the silanized PC/TiO_2_ sample. The results are presented in Figure 4. The changes in ellipsometric parameter δΨ after BSA/anti-BSA complex formation obtained for p-polarization on PC/TiO_2_ was 8° (Figure 4A). The δΔ for the same p-polarization was 74.7° (Figure 4B). The shift in spectrum was δλ_p-BSW_ = 0.8 nm after BSA/anti-BSA complex formation. The change in parameter Ψ after BSA/anti-BSA complex formation (Figure 4C) for the s-polarization component was δΨ_s-BSW_ = 3.9°. The δΔ for the s-polarization component of BSW (Figure 4D) was δΔ_s-BSW_ = 19.1° after BSA/anti-BSA complex formation, and the shift in spectrum was 0.6 nm.

The change in ellipsometric parameter δΨ after BSA/anti-BSA complex formation obtained for p-polarization on PC/TiO_2_ was 8° (Figure 4A). The δΔ for the same p-polarization was 74.7° (Figure 4B). The shift in spectrum was δλ_p-BSW_ = 0.8 nm after BSA/anti-BSA complex formation. The change in parameter Ψ after BSA/anti-BSA complex formation (Figure 4C) for the s-polarization component was δΨ_s-BSW_ = 3.9°. The δΔ for the s-polarization component of BSW (Figure 4D) was δΔ_s-BSW_ = 19.1° after BSA/anti-BSA complex formation and the shift in spectra was 0.6 nm. The sensitivity of both the PC and PC/TiO_2_ samples was evaluated based on the change in the ellipsometric parameters Ψ and Δ at 70° AOI (Figure 3 and Figure 4) divided by the spectral shift (δΨ/δλ and δΔ/δλ). The resolution of the ellipsometer was equal to 1.5 nm; thus, narrow BSW excitations can have distorted spectra. For this reason, the central wavelengths of the BSW excitations are presented as dotted lines (black for PBS, red for BSA and blue for anti-BSA) in Figure 3 and Figure 4. The values of sensitivity parameters for both the p- and s-polarization components of the BSW excitation were evaluated after BSA immobilization and are presented in Table 1. When comparing the sensitivities of the PC and PC/TiO_2_ samples for p- and s-polarizations, it can be seen that the additional TiO_2_ layer enhances the sensitivity of p- and s-polarization in the PC/TiO_2_ sample. However, a much larger enhancement can be seen for the p-polarization component, as the Ψ parameter showed five times higher sensitivity in the PC/TiO_2_ sample than PC and eight times higher sensitivity for the Δ parameter. Meanwhile, for the s-polarization, the Δ sensitivity parameter for the PC sample was three times higher than PC/TiO_2_. This can be explained by the higher quality factor (Q-factor), which showed higher quality of BSW resonance for longer wavelengths compared with p-polarized BSW excitation. These results show an increased sensitivity of p-polarization BSW excitation in a sample with an additional TiO_2_ layer on top of PC. The additional TiO_2_ layer reduces the losses especially for the p-polarization component, as a result reducing the width of the resonance and increasing the sensitivity of the p-BSW because the p-component lies in the plane of incidence, and thus it interacts more with the structure of the additional TiO_2_ layer compared to the s-polarization component, which is reflected from the first interface.

This is also confirmed by the estimation of the Q-factor, which is calculated from the full width at half maximum (FWHM) divided by the central frequency of the resonance. The FWHM was equal to 4.3 nm and 3.6 nm for p-BSW in PC and PC/TiO_2_ samples, respectively, and 3.9 nm and 3.6 nm for s-BSW in PC and PC/TiO_2_ samples, respectively. The quality factor was equal to (Q_p-BSW_)_PC_ = 92.4, (Q_p-BSW_)_PC/TiO2_ = 118.5, (Q_s-BSW_)_PC_ = 155.4, (Q_s-BSW_)_PC/TiO2_ = 173.5. It can be seen that the Q-factor is higher for the sample with additional TiO_2_.

**Figure 5:**
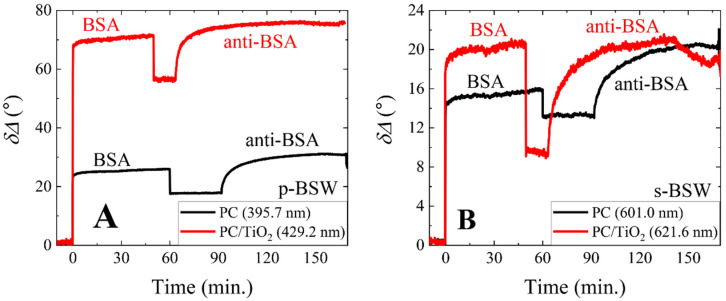
Real-time monitoring of ellipsometric parameter δΔ vs. time: covalent BSA immobilization and BSA/anti-BSA complex formation on the silanized PC sample (black curves) and on the PC/TiO_2_ sample (red curves). (**A**) represents p-polarized BSW, while (**B**) represents BSW excited by s-polarization.

Using the TIRE method, it is possible to obtain the biomolecules’ interaction kinetics for both p- and s-polarizations in real time; such kinetics for covalent BSA immobilization and the BSA/anti-BSA complex are presented in Figure 5. As can be seen from Figure 5A, the δΔ vs. time for covalent BSA immobilization and the formation of the BSA/anti-BSA complex as higher than when using the PC sample. The resonant wavelength for BSW on PC using p-polarized light was 395.7 nm, and on PC/TiO_2_ it was 429.2 nm. These wavelengths were chosen for monitoring of the covalent BSA immobilization and BSA/anti-BSA complex formation. The δΔ for the p-polarization component of BSW excitation after covalent BSA immobilization on PC was 26.48°, and on the PC/TiO_2_ sample it was 71.5°. After BSA/anti-BSA complex formation, the δΔ was 13.34° and 19.77° on PC and PC/TiO_2_ samples, respectively. Figure 5B shows δΔ vs. time for covalent BSA immobilization and the formation of the BSA/anti-BSA complex on PC and PC/TiO_2_ for the BSW s-polarization component. As was previously explained, the PC/TiO_2_ sample’s sensitivity for the monitoring of BSA covalent immobilization is greater than that of the PC sample. The δΔ after BSA covalent immobilization was 15.88° for PC and 20.76° for PC/TiO_2_. After the BSA/anti-BSA complex formation on PC, δΔ was 7.24°, and on PC/TiO_2_ it was 11.87°, respectively.

## 4. Conclusions

One-dimensional PC with an additional TiO_2_ layer, supporting BSW, was employed for enhanced signal sensitivity for the detection of protein interactions. To compare the optical response of BSW, a bovine serum albumin and antigen bovine serum albumin complex was formed. The optical response of BSW excitation was compared for both p- and s-polarization components. The results obtained show the enhanced sensitivity of the p- and s-polarization BSW components for the 1D PC sample with an additional TiO_2_ layer. In particular, a higher sensitivity was obtained for the p-polarization BSW component in a PC sample with an additional TiO_2_ layer, where the sensitivity of the ellipsometric parameter Ψ was five times higher and the Δ parameter was eight times higher than those in the PC sample. This enhancement can be explained by the reduced energy losses in the additional TiO_2_ sample due to the higher amplitude of the electric field at the interface and as a result of the better values of the Q-factor. This shows a possible application of modified PC structures with an additional TiO_2_ layer for enhanced BSW sensitivity in biosensing applications.

## Figures and Tables

**Table 1:** Difference in ellipsometric parameters Ψ and Δ (δΨ, δΔ), shift in spectra (δλ) and sensitivity parameters (δΨ/δλ, δΔ/δλ) for the p- and s-components of BSW in PC and PC/TiO_2_ samples after BSA/anti-BSA complex formation. PC (p-BSW) and PC (s-BSW) indicate the p- and s-polarization components of the BSW for PC sample, respectively. PC/TiO_2_ (p-BSW) and PC/TiO_2_ (s-BSW) indicate the p- and s-polarization components of BSW for PC/TiO_2_ sample.

	δΨ (°)	δΔ (°)	δλ (nm)	(δΨ/δλ) (°/nm)	(δΔ/δλ) (°/nm)
PC (p-BSW)	4.7	30.1	2.5	1.88	12
PC (s-BSW)	1.6	33.1	0.4	4	82.75
PC/TiO_2_ (p-BSW)	8	74.7	0.8	10	93.4
PC/TiO_2_ (s-BSW)	3.9	19.1	0.6	6.5	31.8

## Data Availability

Not applicable.
